# Assessment of Diagnostic and Prognostic Role of Copeptin in the Clinical Setting of Sepsis

**DOI:** 10.1155/2016/3624730

**Published:** 2016-06-06

**Authors:** Stefania Battista, Umberto Audisio, Claudia Galluzzo, Matteo Maggiorotto, Monica Masoero, Daniela Forno, Elisa Pizzolato, Marco Ulla, Manuela Lucchiari, Annarita Vitale, Corrado Moiraghi, Enrico Lupia, Fabio Settanni, Giulio Mengozzi

**Affiliations:** ^1^Emergency Medicine Department, “Città della Salute e della Scienza” University Hospital of Turin, Corso Bramante 88, 10126 Turin, Italy; ^2^Clinical Biochemistry Laboratory, “Città della Salute e della Scienza” University Hospital of Turin, Corso Bramante 88, 10126 Turin, Italy

## Abstract

The diagnostic and prognostic usefulness of copeptin were evaluated in septic patients, as compared to procalcitonin assessment. In this single centre and observational study 105 patients were enrolled: 24 with sepsis, 25 with severe sepsis, 15 with septic shock, and 41 controls, divided in two subgroups (15 patients with gastrointestinal bleeding and 26 with suspected SIRS secondary to trauma, acute coronary syndrome, and pulmonary embolism). Biomarkers were determined at the first medical evaluation and thereafter 24, 48, and 72 hours after admission. Definitive diagnosis and in-hospital survival rates at 30 days were obtained through analysis of medical records. At entry, copeptin proved to be able to distinguish cases from controls and also sepsis group from septic shock group, while procalcitonin could distinguish also severe sepsis from septic shock group. Areas under the ROC curve for copeptin and procalcitonin were 0.845 and 0.861, respectively. Noteworthy, patients with copeptin concentrations higher than the threshold value (23.2 pmol/L), calculated from the ROC curve, at admission presented higher 30-day mortality. No significant differences were found in copeptin temporal profile among different subgroups. Copeptin showed promising diagnostic and prognostic role in the management of sepsis, together with its possible role in monitoring the response to treatment.

## 1. Introduction

Sepsis, severe sepsis, and septic shock are among the leading causes of death in the Emergency Departments and Intensive Care Units [1]. It is estimated that there are more than 1.000.000 cases of sepsis among hospitalized patients each year in USA [2]. Prompt diagnosis and therapy administration are considered key features to improve septic patient outcomes as indicated in the most recent sepsis guidelines published in 2013 by Surviving Sepsis Campaign [3], even if the EGDT (Early Goal-Directed Therapy) protocol efficacy has been questioned by several studies [4, 5]. The use of biomarkers may be useful in the detection of infection and in the management of the septic patient; despite the fact that more than 100 molecules have been evaluated in sepsis [6], an ideal biomarker, that is to say a molecule which, at the same time, allows early diagnosis, risk stratification, monitoring of clinical response to therapy, and prediction of outcome [7], is still missing. Procalcitonin (PCT) is the reference biomarker in the diagnosis of sepsis: plasma levels more than two standard deviations above the normal value are part of the sepsis definition [3]. Moreover, PCT may be useful in helping the physician in the decision of discontinuing the antibiotic therapy therefore limiting antibiotic overuse [8]. Copeptin is a 39-amino acid glycopeptide derived from preprovasopressin and it is cosecreted in the bloodstream with vasopressin in an equimolar ratio in response to osmotic and hemodynamic changes. It is slowly degraded and in healthy individuals normal values of copeptin range between 1.70 and 11.25 pmol/L [9]. Copeptin physiological role(s) in the circulation is not yet known; nevertheless it is used in routine assessment instead of vasopressin, because of its easier measurability. As a matter of fact copeptin has been used to evaluate the role of vasopressin in pathophysiology [10]; its measurement proved to be useful as a novel approach for diabetes insipidus (DI) diagnosis, allowing the distinction between central complete and nephrogenic DI. Moreover, increased level of copeptin is related to higher mortality in patients with chronic and acute heart failure. In the study by Voors et al. [11] copeptin is shown to be a stronger biomarker in prediction of death and cardiovascular events than both BNP (brain natriuretic peptide) and NT-pro-BNP (N-terminal pro-BNP) in a population of patients with heart failure after acute myocardial infarction. Clinical conditions like severe sepsis and septic shock are responsible for a relevant secretion of copeptin and vasopressin, causing a function impairment of the latter. This impairment is thought to be partially responsible of human body's inability to reestablish a correct vascular tone in the patient suffering from septic shock [12]. Based on these premises, we compared copeptin with PCT in terms of diagnostic and prognostic efficacy in an adult population admitted to the Emergency Department (ED) with SIRS (systemic inflammatory response syndrome) or active gastrointestinal bleeding (control group) and suspected sepsis, severe sepsis, and septic shock (study group).

## 2. Materials and Methods

### 2.1. Study Design and Patients

We conducted a single centre and observational study on patients presenting to ED with diagnosis of gastrointestinal bleeding, SIRS and either confirmed or suspected sepsis, severe sepsis, and septic shock at the first medical evaluation. Patient enrollment was conducted in an eight-month period. An informed consent to participate in the study, in accordance with the principles of Ethics Committee of Turin University Hospital based on the Declaration of Helsinki (1964), was obtained from each patient. Sixty-four patients (26 females and 38 males) were referred to the ED of Città della Salute e della Scienza University Hospital of Turin with the suspicion of sepsis, severe sepsis, or septic shock and forty-one subjects (13 females and 28 males) affected by SIRS or gastrointestinal bleeding were enrolled as cases and controls, respectively. The inclusion criteria were patients who could undergo a blood test at the admission (T0) and thereafter at 24 (T1), 48 (T2), and 72 (T3) hours after enrollment, presence of at least two clinical criteria for SIRS, suspected diagnosis of sepsis, severe sepsis, and septic shock (according to the American College of Chest Physicians/Society of Critical Care Medicine and the International Surviving Sepsis Campaign Guidelines Committee) [3], and patients with diagnosis of gastrointestinal (GI) bleeding at first medical evaluation. Exclusion criteria were patients unable to provide informed legal consent, age less than 18 years, patients with previous diagnosis of endocrine disease involving the hypothalamic-pituitary gland axis, and patients dismissed or deceased with new diagnosis of endocrine disease involving the hypothalamic-pituitary gland axis. On admission to the ED, clinical and biochemical data were collected before any medical treatment; using clinical parameters and blood test results, Simplified Acute Physiology Score (SAPS II) [13] and Sequential Organ Failure Assessment (SOFA) score [14] were calculated. For the gastrointestinal bleeding control group also the Blatchford score has been calculated [15]. In Table 1 demographic and clinical characteristics of the recruited patients are shown. The analysis of digital medical records and the criteria of the International Guidelines for Management of Severe Sepsis and Septic Shock [3] were used to obtain definitive diagnosis (SIRS, sepsis, severe sepsis, septic shock, and GI bleeding); the prognostic role of copeptin was assessed by correlating its values to the in-hospital mortality at 30 days.

### 2.2. Biomarker Measurements

Collected samples were stored at −70°C and were subsequently analyzed for procalcitonin and copeptin in the Clinical Biochemistry Laboratory. Copeptin concentrations were determined using the BRAHAMS KRYPTOR compact PLUS automated method, which requires 20 minutes to be completed. It is a quantitative test that allows the assessment of CT-pro-AVP (C-terminal portion of proarginine-vasopressin or copeptin) concentrations in human serum or plasma (EDTA, heparin) by time-resolved amplified cryptate emission (TRACE) technique which measures the signal that is emitted from an immunocomplex with time delay. The assay has a functional sensitivity of 0.9 pmol/L; imprecision evaluation tests yielded a within-run variation of less than 7% and a between-run variation of less than 12% on a wide range of values. Procalcitonin concentrations were measured by the method Elecsys® BRAHMS PCT (Roche Diagnostics). It is an electrochemiluminescence immunoassay (ECLIA) for the in vitro quantitative determination of the concentration of procalcitonin (PCT) in serum or plasma. The test took about 18 minutes and the assays were performed automatically on the analyzer e601® Cobas (Roche Diagnostics, Mannheim, Germany). Within-series and between-series coefficient of variations were <6% and <13% on different sample concentrations, and the functional sensitivity has been calculated <0.02 ng/mL.

### 2.3. Statistical Analysis

The One-Way Analysis of Variance (ANOVA) followed by Student-Newman-Keuls *t*-test was used to evaluate differences among the five patient populations and to compare the diagnostic efficacy of biomarkers in the study. The diagnostic accuracy at admission of study biomarkers was evaluated through the analysis of the ROC (Receiving Operator Characteristic) curves. The prognostic role of copeptin values at entry was estimated through Kaplan-Meier curve. Serial measurement analysis was applied to assess differences in the areas under the curve of biomarker temporal profiles over a 72-hour interval. Both within- and between-group differences among biomarker concentrations detected at admission and thereafter at 24, 48, and 72 hours were tested by ANOVA for repeated measures. Statistical analysis and graphing were performed using the software MedCalc® (MedCalc Software, Belgium). A *p* value < 0.05 was considered to be significant.

## 3. Results

**Figure 1:**
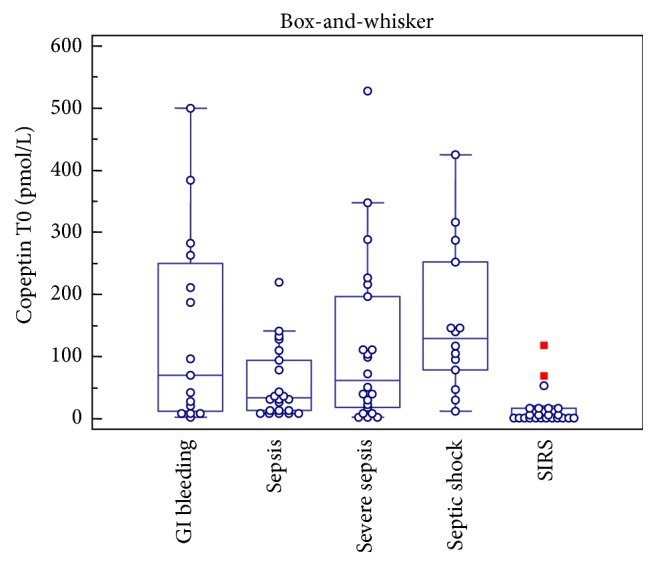
Distribution of copeptin concentrations obtained at admission (T0) in the studied groups. Box-and-whisker plot represents median, first, and third quartile, as well as minimum and maximum values.

The study was carried out on a total of 105 patients: 15 were diagnosed with gastrointestinal bleeding, 26 were suffering from a condition of SIRS without evidence of infectious outbreak, 24 were diagnosed with sepsis, 25 were diagnosed with severe sepsis, and 15 were diagnosed with septic shock. The ANOVA test proved to be significant for both copeptin and PCT. The median copeptin concentrations were 70.1 pmol/L (range 2.95–500.1) in the gastrointestinal bleeding group, 5.2 pmol/L (1.25–117.8) in the SIRS group, 34.2 pmol/L (7.9–220.1) in the sepsis group, 61.8 pmol/L (2.5–527.7) in the severe sepsis group, and 128.7 pmol/L (12.0–425.0) in the septic shock group (Figure 1).

**Figure 2:**
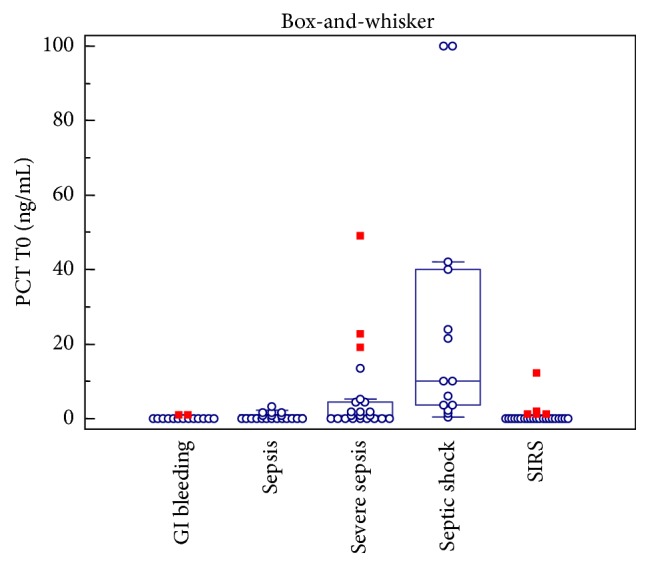
Distribution of procalcitonin (PCT) concentrations measured at entry (T0) in the studied groups. Box-and-whisker plot represents median, first, and third quartile, as well as minimum and maximum values.

Upon arrival to the Emergency Department, copeptin concentrations were significantly higher in patients with septic shock than in those with sepsis (*p* = 0.02); moreover copeptin concentrations were significantly different between SIRS group and septic population (*p* < 0.05). The gastrointestinal bleeding controls showed very high concentrations of copeptin but no statistically significant difference has been observed between this group and septic population (*p* > 0.05). Interestingly, C-reactive protein (CRP) concentrations were very similar in the three populations of septic patients, and no statistically significant differences were found among studied groups except for the gastrointestinal bleeding controls, presenting with lower values. Accordingly, no correlations were observed between copeptin and PCR levels in our study. PCT concentrations in gastrointestinal bleeding, SIRS, sepsis, severe sepsis, and septic shock groups were 0.15 ng/mL (0.02–1.03), 0.03 ng/mL (0.02–12.3), 0.39 ng/mL (0.04–3.21), 0.74 ng/mL (0.08–49.11), and 10.10 ng/mL (0.34–100.00), respectively (Figure 2).

PCT concentrations were similar in SIRS and gastrointestinal bleeding groups (*p* = 0.2772). The biomarker distinguished the septic population from controls (*p* < 0.05), and its concentrations were significantly different among sepsis, severe sepsis, and septic shock groups.

**Figure 3:**
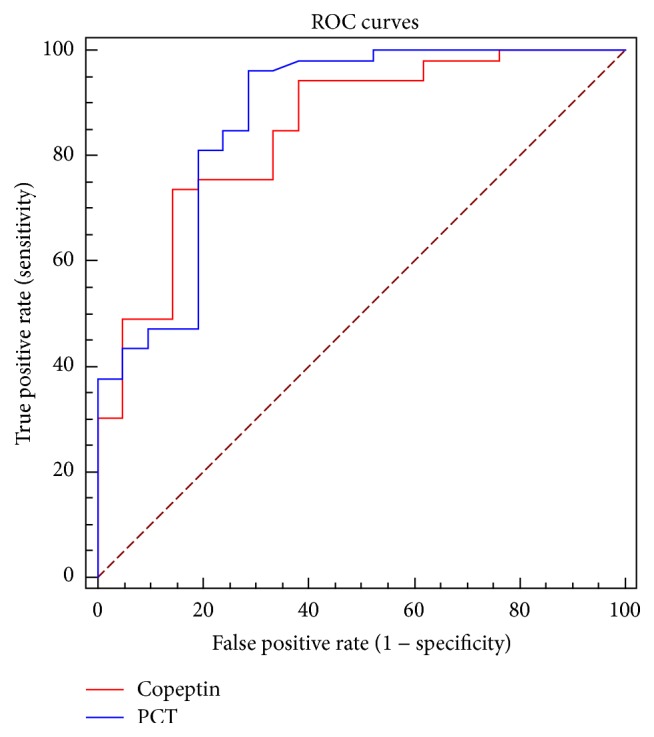
Comparison between ROC curves obtained for copeptin and procalcitonin (PCT) on samples collected at the admission to the Emergency Department.

The analysis of the diagnostic role of the two biomarkers was focused only on the four groups of patients with suspected sepsis or septic shock. The ROC curve of copeptin shows an AUC of 0.845. The best cut-off in terms of diagnostic accuracy was found at a copeptin concentration of 23.2 pmol/L, with 74% sensitivity and 87% specificity. The AUC for PCT ROC curve was 0.861 and the best cut-off in terms of diagnostic accuracy was found at PCT levels of 0.090 ng/mL, with 96% sensitivity and 73% specificity. A statistically significant difference between the ROC curves of copeptin and PCT was not observed (*p* = 0.85) (Figure 3).

**Figure 4:**
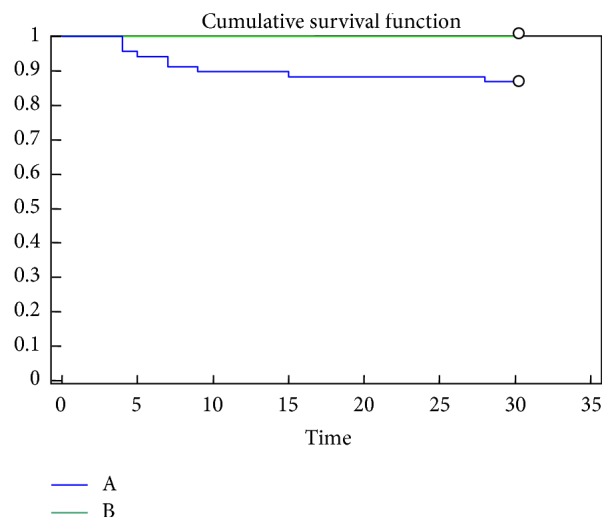
Kaplan-Meier survival curves related to copeptin concentrations at admission to the Emergency Department. A: patients with initial copeptin concentrations higher than 23.2 pmol/L; B: patients with copeptin concentrations at entry lower than 23.2 pmol/L.

Regarding the prognostic effectiveness of copeptin, placing as limit the value of 23.2 pmol/L, obtained from the analysis of the ROC curve, and considering the deaths up to 30 days, it can be deduced that individuals with concentrations of copeptin on admission higher than the threshold value were burdened by a higher mortality (*p* = 0.047) (Figure 4).

By comparing the area under the curve of temporal profiles of the two biomarkers, until 72 hours after enrollment, serial measurement analysis showed some differences among the populations with sepsis, severe sepsis, and septic shock. As regards copeptin, this difference did not reach statistical significance; however, it was possible to observe a different trend among the patients' populations, particularly among patients diagnosed with sepsis and those diagnosed with severe sepsis or septic shock. The temporal profile of PCT concentrations, on the other hand, showed a significant difference between the three populations, in particular between sepsis and septic shock, demonstrating the utility of PCT in monitoring the evolution of the clinical response to treatment. These observations were confirmed by the ANOVA for repeated measures, demonstrating a statistically significant reduction in PCT concentrations from T0 to T72 (*p* < 0.0001), with significant differences both within- and between-groups. The same analysis performed with copeptin results revealed only small between-group differences in biomarker fluctuations over time, even if more pronounced and near the statistical significance for the high risk group of septic shock patients.

## 4. Discussion

Sepsis, which is a systemic inflammatory response secondary to infection, is responsible for an endocrine dysfunction characterized by inappropriately low levels of vasopressin, sick euthyroid syndrome, reduced adrenal response to ACTH, insulin resistance, and hyperglycemia [16]. In the early stages of septic shock, sepsis-induced hypotension is one of the main stimuli of vasopressin secretion, causing an increase in the hormone serum levels which contributes to the maintenance of arterial pressure [17]; subsequently a rapid decline is observed, due to vasopressin stores depletion. Vasopressin function impairment is one of the many factors leading to development of the decrease of arterial pressure [12] and prolonged hypotension and the resulting hypoperfusion leads to organ failure; maintenance of and adequate level of mean arterial pressure (MAP) is a main goal in the management of septic shock [18]. Unfortunately, reliable measurement of plasma vasopressin concentrations is difficult due to its short half-life (24 minutes), its small size, and the complex preanalytical steps required [9]. Copeptin, which is the C-terminal fragment of provasopressin peptide (CT-proAVP) and is coreleased in an equimolar ratio with vasopressin, instead, is a stable peptide in EDTA plasma and can be used as a surrogate biomarker of arginine vasopressin [10]. Copeptin has been evaluated as biomarker for several illnesses such as stroke [19] or heart failure [20], showing a promising role mainly as a prognostic biomarker. In a recent study copeptin levels seem to be strongly related to short-, mid-, and long-term mortality in unselected patients admitted to hospital showing that copeptin could be a valuable prognostic tool in the most frequent disease entities [21]; moreover, together with other stress markers, it could be used in risk stratification of patients presenting to the ED with nonspecific complaints [22].

Several studies that highlight the role of copeptin as prognostic factor of increased risk in sepsis and septic shock have also been published. Copeptin levels increase progressively with the severity of sepsis in ventilator associated pneumonia (VAP) [23], copeptin plasma concentrations in patients with sepsis are positively related to APACHE II score and reflect disease severity [24], and elevated copeptin levels in patients with septic or haemorrhagic shock [25] predict poor outcomes.

With the aim to verify whether copeptin could be useful as a biomarker of sepsis we designed a single centre study in which we evaluated the role of copeptin compared to PCT in the management of patients referred to the ED of a tertiary care referral teaching hospital with suspected sepsis, severe sepsis, and septic shock. Overall, we evaluated 105 patients divided in four groups according to the definition criteria of SIRS, sepsis, severe sepsis, and septic shock validated by the most recent International Guidelines. The control group population included subjects with SIRS and also subjects with active gastrointestinal (GI) bleeding. The choice of including patients with GI bleeding in the control group was made in order to evaluate copeptin trend in those patients with an hemodynamic alteration due to an hypovolemic and not distributive stress. For both biomarkers we determined diagnostic accuracy on admission, as well as temporal profile in blood concentrations at 24, 48, and 72 hours after admission. Prognostic effectiveness of copeptin concentrations related to mortality at 30 days after enrollment was also evaluated.

Our data suggest a potential use of copeptin as diagnostic tool in a population of patients referred to the ED with suspected sepsis. In this study copeptin is shown to be superior to CRP in distinguishing the population of patients diagnosed with septic shock from those diagnosed with sepsis. Copeptin secretion is associated with a reactive response to important changes in blood volume, as seen in septic and haemorrhagic shock [25]; in the present study the septic shock group and the control group of patients with GI bleeding have high levels of copeptin plasma concentrations. It cannot be excluded, however, that proinflammatory stimuli and activation of the immune system can induce the increase of copeptin concentrations in septic shock, as in the case of procalcitonin, since it has been shown that inflammatory mediators such as interleukin-1*β* (IL-1*β*) and Tumor Necrosis Factor-*α* (TNF*α*) stimulate the secretion of AVP and copeptin [26].

The threshold limit value calculated in our study (23.2 pmol/L) differs from the one proposed by Morgenthaler et al. (96 pmol/L) [25]. This difference could be due to different criteria used in patient enrollment and to sample size.

In the analysis of the potential benefits deriving from the use of copeptin in predicting the outcomes of a population of critically ill patients, the role of this biomarker as prognostic index on survival cannot be overlooked, already documented, and confirmed also in this study. In fact, our observations prove that patients with lower concentrations of copeptin on admission have a lower probability of dying. It could be useful to evaluate copeptin as a prognostic factor for other endpoints different from mortality (length of hospitalization, length of treatment with elevated costs, and long-term sepsis-induced organ damage) and as an independent prognostic index when compared with other variables related to risk (clinical scores, biohumoral values like lactate levels, and hemodynamic parameters).

In this regard, the assessment of the temporal profile of the two biomarkers concentrations in the first 72 hours after admission has been performed. This analysis showed, in agreement with data reported in the literature [27, 28], the utility of PCT in monitoring the evolution of the clinical response to treatment, particularly for the population of patients diagnosed with severe sepsis or septic shock, which present a significantly different trend of serial concentrations when compared to the population of subjects with a diagnosis of sepsis. Copeptin showed a similar behaviour, although not reaching statistical significance a different trend is observed in the group of patients diagnosed with sepsis, suggesting a possible application of copeptin also in monitoring the clinical response to treatment in these populations.

To the best of our knowledge, a multibiomarker approach that involves both PCT and copeptin needs to be evaluated yet. When compared to classical inflammation biomarkers [29], like PCT, copeptin seems to have a lower diagnostic capacity. Nevertheless, considering the differences in the induction of both biomarkers in a complex pathophysiological contest such as that represented by the continuum of sepsis, an approach based on a combined use of the two biomarkers should be evaluated on a larger sample size in order to assess the degree of additional information and the impact on clinical decision-making that this approach could offer. In such assessment a careful examination of the cost benefit ratio must be inevitably included.

## 5. Conclusions

With this study we demonstrate the potential role of copeptin in the management of patients referring to the Emergency Department with suspected sepsis in three different areas of application: diagnosis, prognosis, and monitoring. Results related to diagnostic potential of copeptin show that this biomarker is able to differentiate between the populations and stratify the disease severity, in particular patients diagnosed with sepsis from patients diagnosed with septic shock. As prognostic index, lower concentrations of copeptin on admission are related to lower mortality. Unlike PCT, copeptin levels monitoring is not statistically significant, although a different trend is observable between the sepsis group and the severe sepsis and septic shock groups. Presented data highlight the need to investigate and confirm the diagnostic and prognostic role of copeptin, as well as its possible use in the monitoring of the disease evolution of patients with suspected sepsis, with further studies based on larger sample size.

## Figures and Tables

**Table 1:** Demographic and clinical characteristics of the study population.

	Controls (*N* = 41)	Sepsis (*N* = 24)	Severe sepsis (*N* = 25)	Septic shock (*N* = 15)
	SIRS (*N* = 26) GI bleeding (*N* = 15)			
Females (*N*)	28	11	16	11
Males (*N*)	13	13	9	4
Age (years)	53.4 (18–92)	67.1 (20–85)	68.8 (50–83)	77.5 (66–93)
SAPSS II	31 (15–54)	28 (13–52)	35 (20–50)	46 (35–60)
SOFA	3 (0–6)	2 (0–7)	3 (0–7)	9 (5–12)
Blatchford	13 (9–18)			

Values are expressed as mean and range. SIRS: systemic inflammatory response syndrome; GI bleeding: gastrointestinal bleeding; SAPS II: Simplified Acute Physiology Score; SOFA score: Sequential Organ Failure Assessment score.
